# Supplementary material for: Evaluating transportability of in vitro cellular models to in vivo human phenotypes using gene perturbation data
Source: Nat Commun. 2025 Dec 13;17:513. doi: 10.1038/s41467-025-67199-1 (PMC12804867; doi:10.1038/s41467-025-67199-1)
Supplement: Supplementary file 1 — Supplementary Information [file 41467_2025_67199_MOESM1_ESM.pdf]

Supplementary Information for "Evaluating transportability of *in-vitro* cellular models to *in-vivo* human phenotypes using gene perturbation data"

Laurence J Howe[1], Yurii S Aulchenko[1], George Davey Smith[2], Neil M Davies[3], Jorge Esparza-Gordillo[4], Toby Johnson[1], Jimmy Z Liu[5], Tom G Richardson[1], Philippe Sanseau[1], Robert A Scott[1], Daniel D Seaton[1], Ashwini Sharma[1], Adrian Cortes[6]

[1] GSK, Gunnels Wood Road, Stevenage, SG1 2NY, United Kingdom
[2] MRC-IEU, University of Bristol, UK
[3] Division of Psychiatry, University College London, UK
[4] GSK, Calle Severo Ochoa, 28760 Tres Cantos, Spain
[5] GSK, Collegeville, Pennsylvania, USA
[6] GSK, Meyerhofstrasse 1, 69117, Heidelberg, Germany

Table of Contents

Supplementary Figures **2**
Supplementary Figure 1 Intracellular insulin content and Hba1c 2
Supplementary Figure 2 Intracellular insulin content and Type II diabetes 2
Supplementary Figure 3 Adipocyte differentiation and body-mass index 4
Supplementary Figure 4 Adipocyte differentiation and waist-circumference 5
Supplementary Figure 5 Adipocyte differentiation and body fat percentage 6
Supplementary Figure 6 GPAT simulations: example of baseline model 7
Supplementary Figure 7 GPAT simulations: example of balanced pleiotropy model 8
Supplementary Figure 8 GPAT simulations: example of unbalanced pleiotropy model 9
Supplementary Figure 9 GPAT simulations: example of phenotypic pleiotropy model 10
Supplementary Figure 10 GPAT simulations: example of directional inconsistency model 11

Supplementary Tables **12**
Supplementary Table 1 GPAT and MR for LDL-C and CHD 12
Supplementary Table 2 Gene-set enrichment estimates 13
Supplementary Table 3 GPAT estimates for lysosomal cholesterol accumulation and LDL-C 14
Supplementary Table 4 GPAT estimates for chondrocyte proliferation and standing height 15
Supplementary Table 5 GPAT estimates for adipocyte differentiation and adiposity-related phenotypes 16
Supplementary Table 6 GPAT estimates for insulin content and diabetes / Hba1c 17
Supplementary Table 7 BioGRID ORCS data 18
Supplementary Table 8 Hypothesis-free GPAT estimates 23
Supplementary Table 9 GPAT simulation results (N = 100) 25
Supplementary Table 10 UK Biobank phenotypes 26

Supplementary Figures

Supplementary Figure 1 Funnel plot for intracellular insulin content and Hba1c

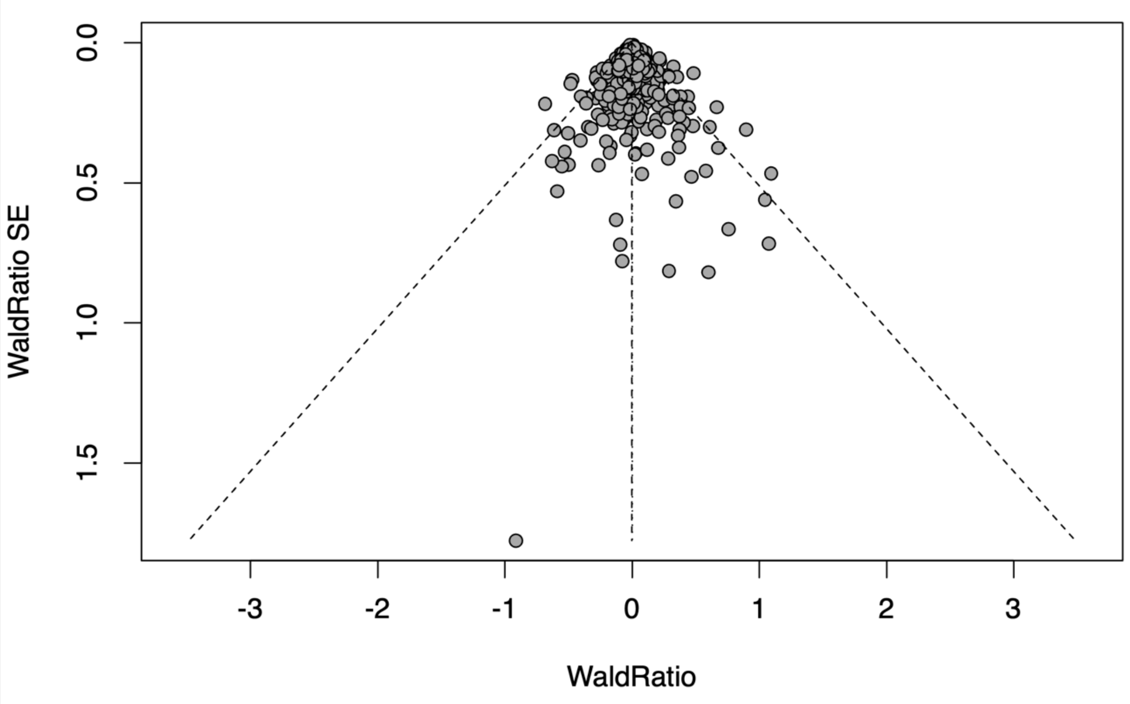

Each data point is the Wald ratio for a gene associated with intracellular insulin content, a point estimate indicating the relationship between intracellular insulin content and Hba1c with the corresponding standard error. The dashed lines represent the fixed-effects (longer bars) and random-effects (shorter bars) meta-analysis estimates and the 95% confidence intervals. Estimates are presented on the linear scale.

Supplementary Figure 2 Funnel plot for intracellular insulin content and Type II diabetes

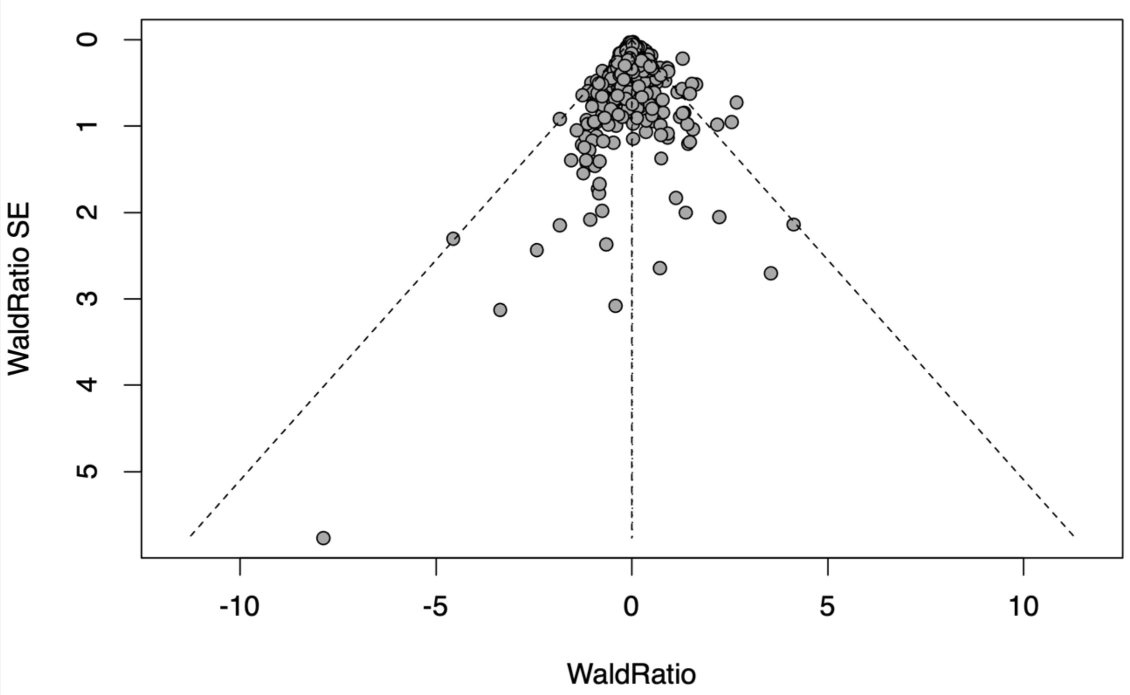

Each data point is the Wald ratio for a gene associated with intracellular insulin content, a point estimate indicating the relationship between intracellular insulin content and type II diabetes with the corresponding standard error. The dashed lines represent the fixed-effects (longer bars) and random-effects (shorter bars) meta-analysis estimates and the 95% confidence intervals. Estimates are presented on the linear scale.

Supplementary Figure 3 Funnel plot for adipocyte differentiation and body-mass index

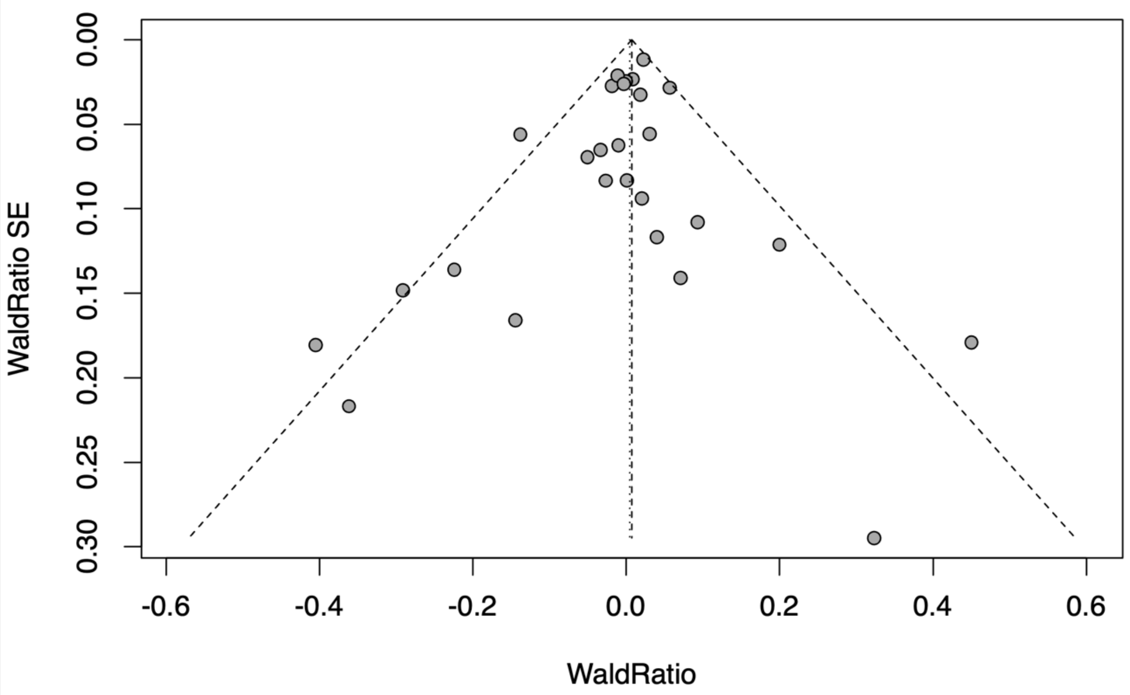

Each data point is the Wald ratio for a gene associated with adipocyte differentiation, a point estimate indicating the relationship between adipocyte differentiation and body-mass index with the corresponding standard error. The dashed lines represent the fixed-effects (longer bars) and random-effects (shorter bars) meta-analysis estimates and the 95% confidence intervals. Estimates are presented on the linear scale.

Supplementary Figure 4 Funnel plot for adipocyte differentiation and waist-circumference

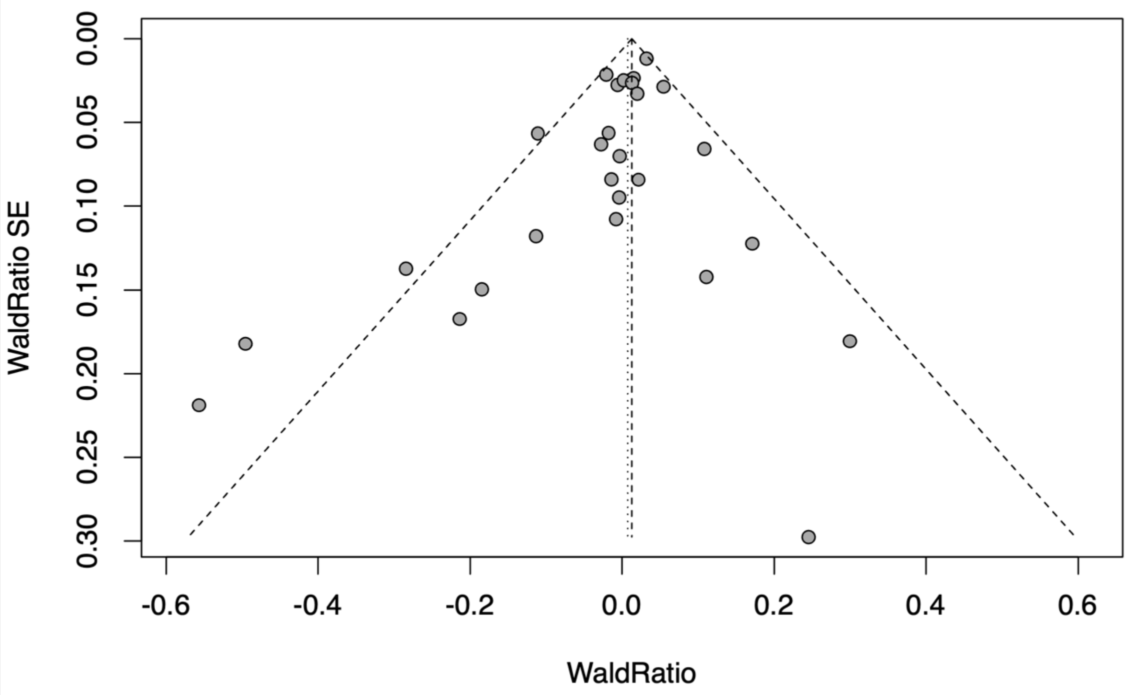

Each data point is the Wald ratio for a gene associated with adipocyte differentiation, a point estimate indicating the relationship between adipocyte differentiation and waist-circumference with the corresponding standard error. The dashed lines represent the fixed-effects (longer bars) and random-effects (shorter bars) meta-analysis estimates and the 95% confidence intervals. Estimates are presented on the linear scale.

Supplementary Figure 5 Adipocyte differentiation and body fat percentage

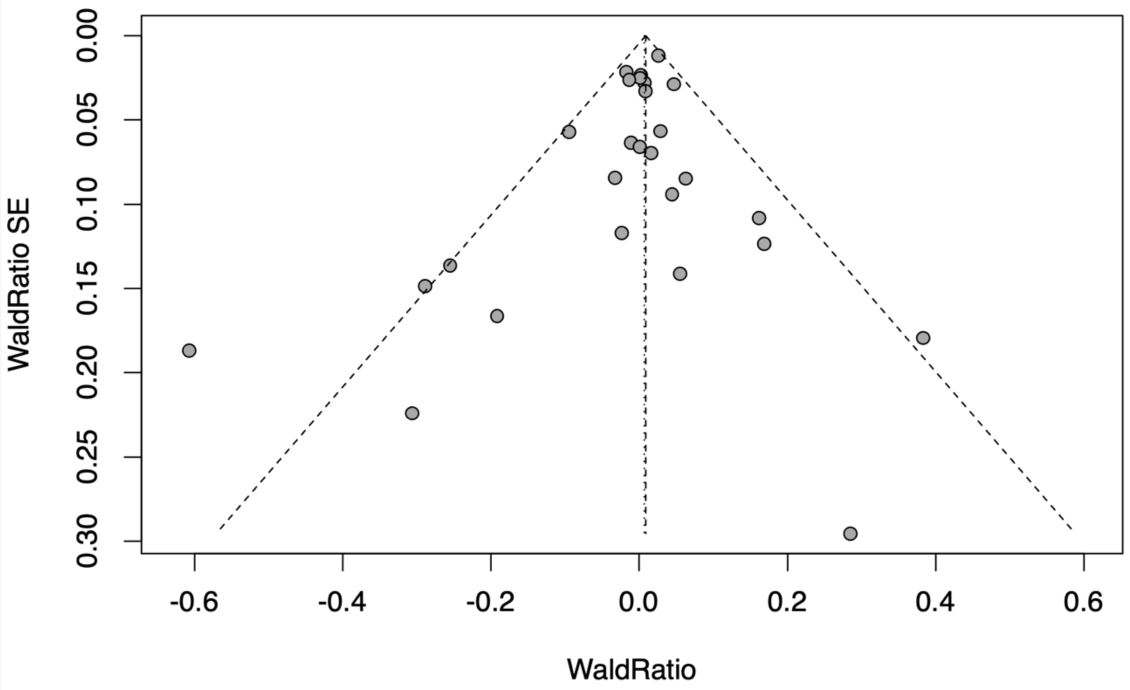

Each data point is the Wald ratio for a gene associated with adipocyte differentiation, a point estimate indicating the relationship between adipocyte differentiation and body fat percentage with the corresponding standard error. The dashed lines represent the fixed-effects (longer bars) and random-effects (shorter bars) meta-analysis estimates and the 95% confidence intervals. Estimates are presented on the linear scale.

Supplementary Figure 6 GPAT simulations: example of baseline model

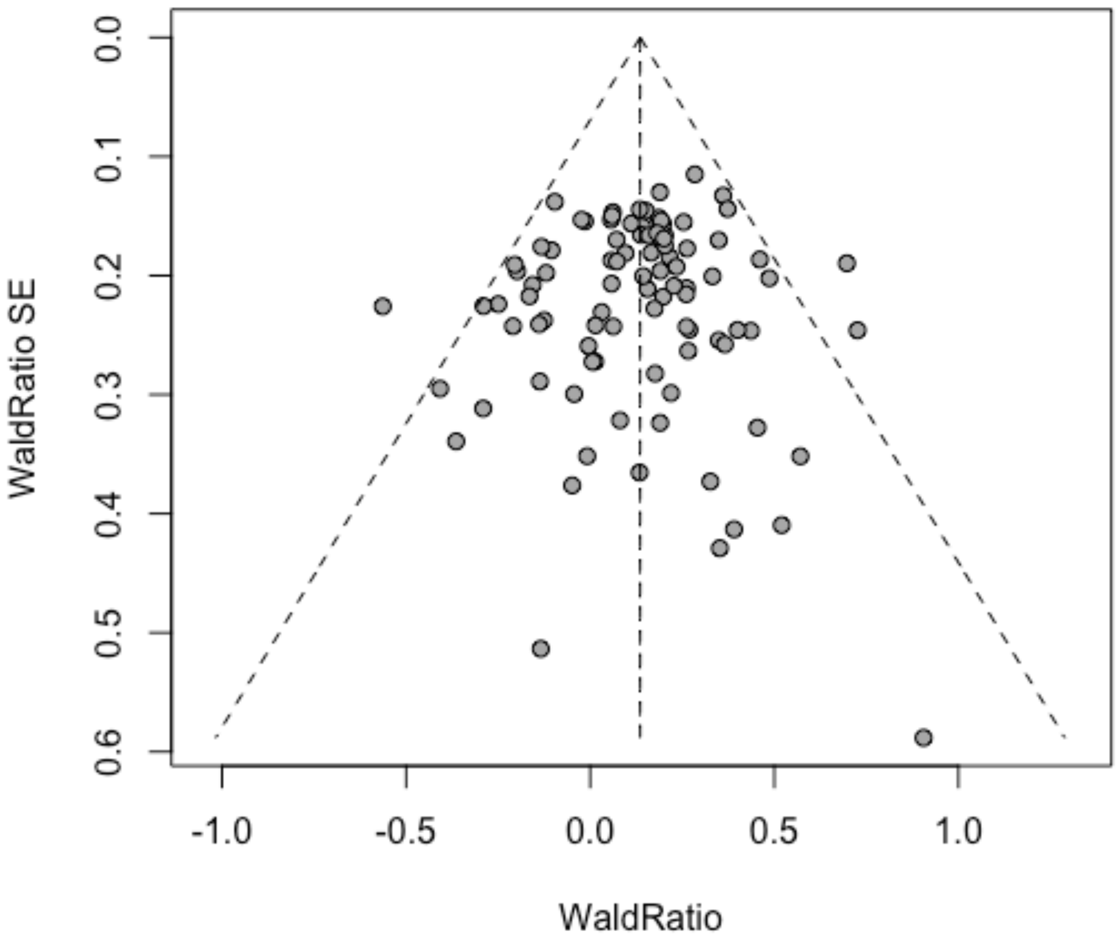

Each data point is the Wald ratio for a gene from simulation analyses under the baseline model. The dashed lines represent the fixed-effects (longer bars) and random-effects (shorter bars) meta-analysis estimates and the 95% confidence intervals. Estimates are presented on the linear scale.

Supplementary Figure 7 GPAT simulations: example of balanced pleiotropy model

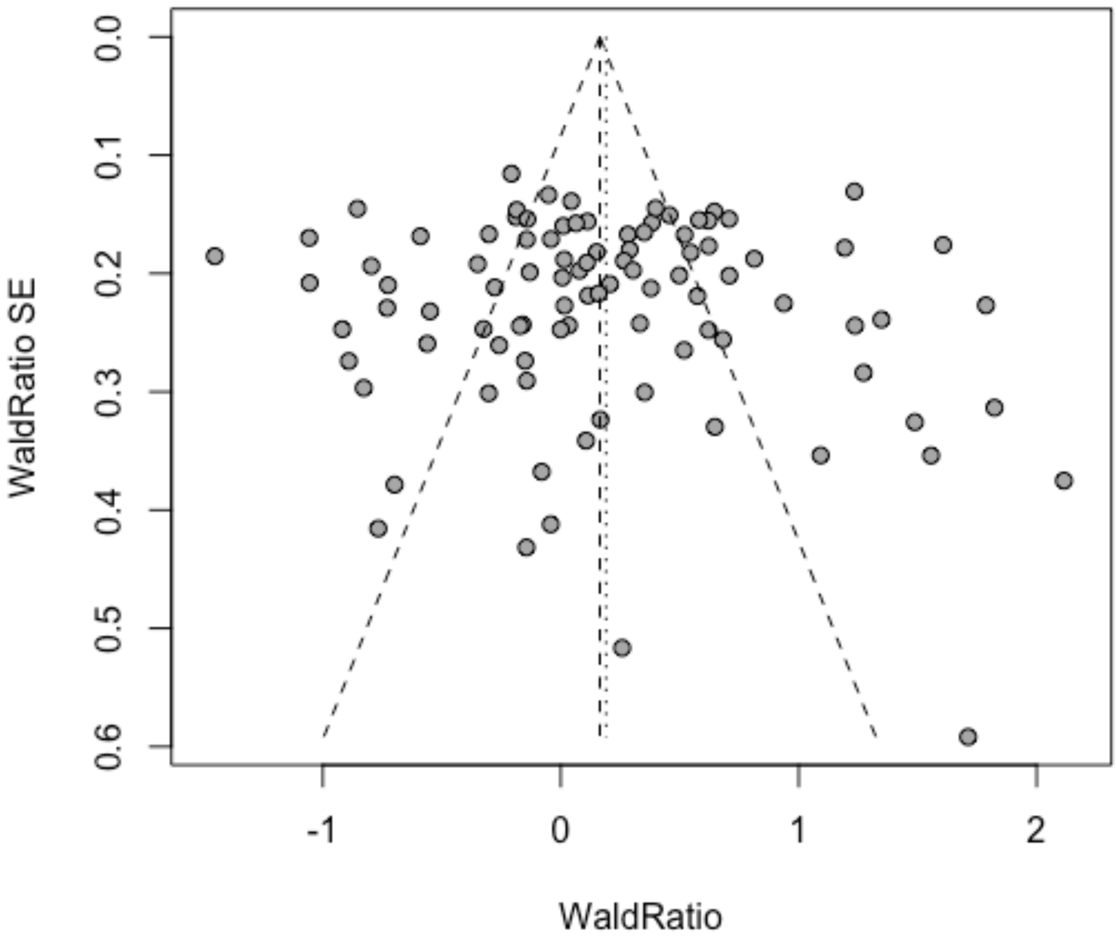

Each data point is the Wald ratio for a gene from simulation analyses under the balanced pleiotropy model. The dashed lines represent the fixed-effects (longer bars) and random-effects (shorter bars) meta-analysis estimates and the 95% confidence intervals. Estimates are presented on the linear scale.

Supplementary Figure 8 GPAT simulations: example of unbalanced pleiotropy model

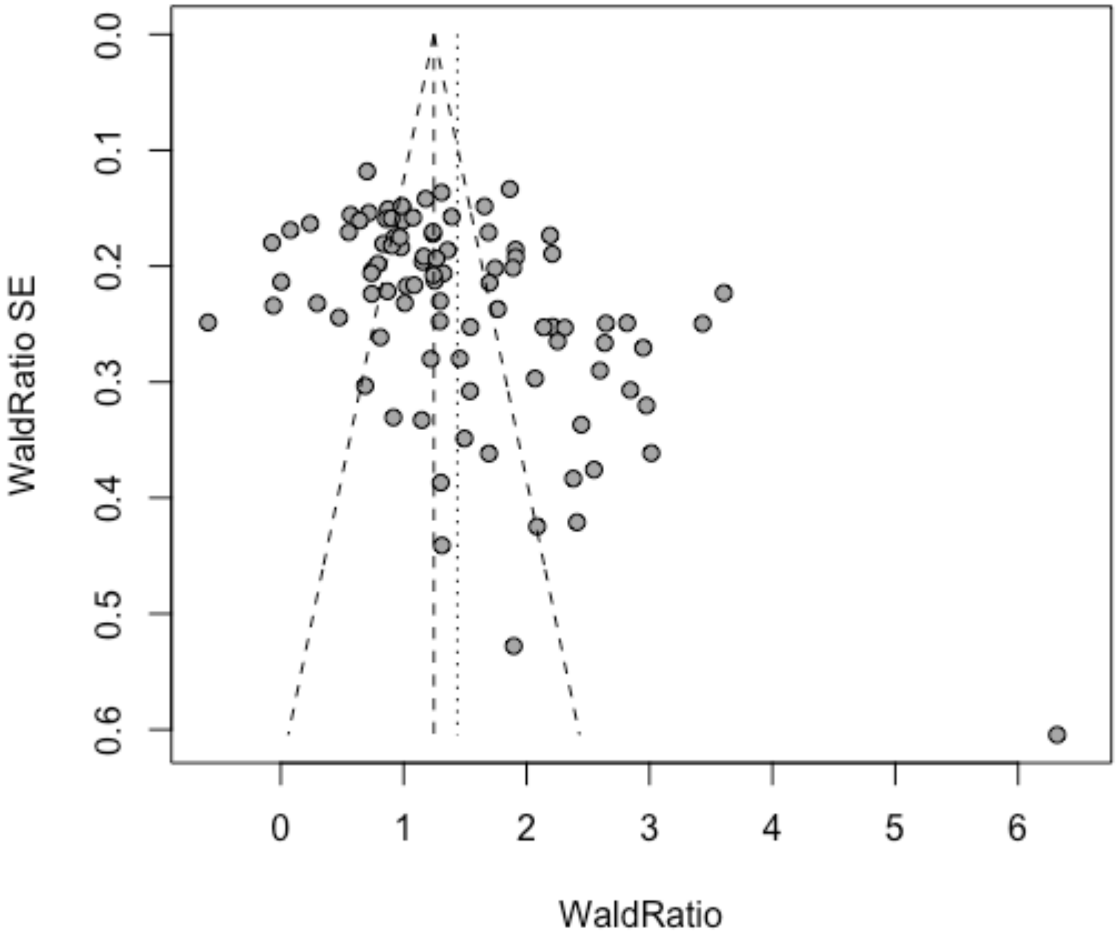

Each data point is the Wald ratio for a gene from simulation analyses under the unbalanced pleiotropy model. The dashed lines represent the fixed-effects (longer bars) and random-effects (shorter bars) meta-analysis estimates and the 95% confidence intervals. Estimates are presented on the linear scale.

Supplementary Figure 9 GPAT simulations: example of phenotypic pleiotropy model

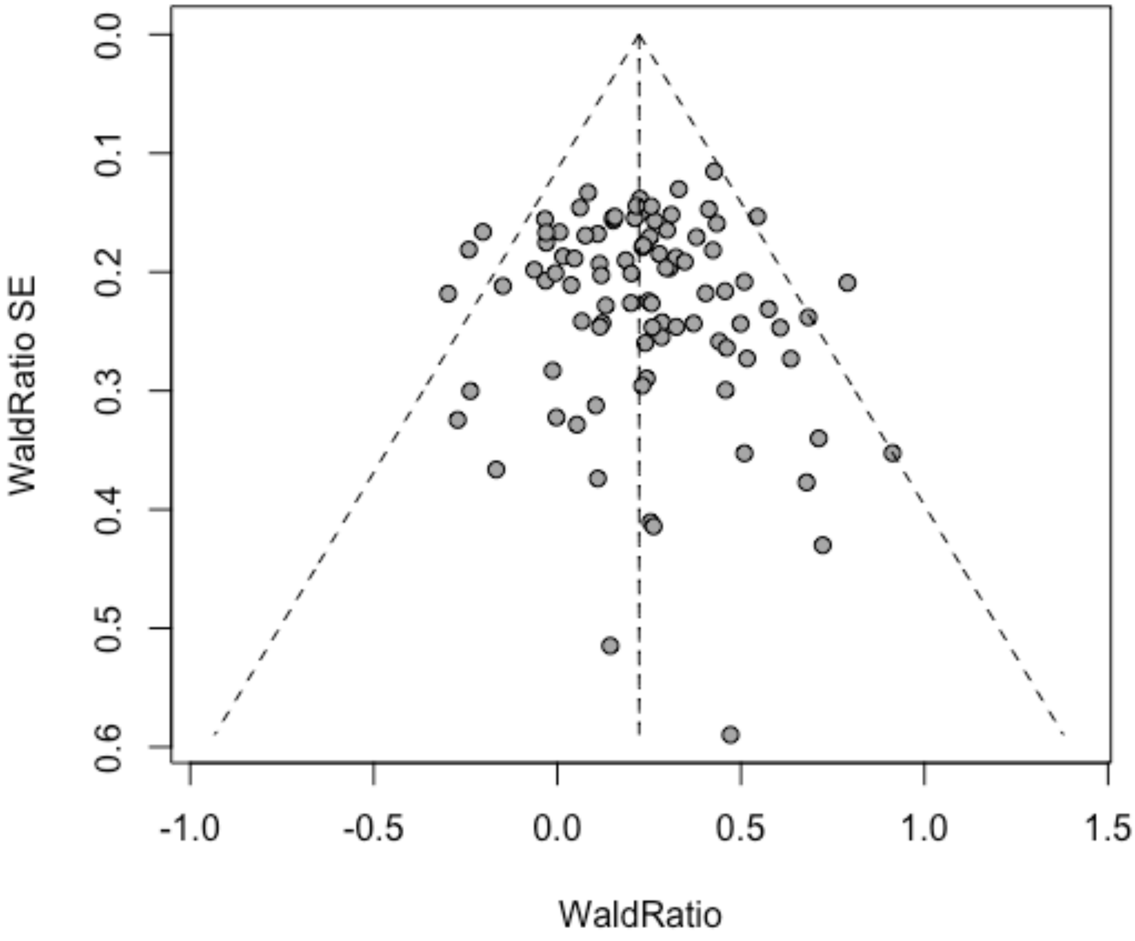

Each data point is the Wald ratio for a gene from simulation analyses under the phenotypic pleiotropy model. The dashed lines represent the fixed-effects (longer bars) and random-effects (shorter bars) meta-analysis estimates and the 95% confidence intervals. Estimates are presented on the linear scale.

Supplementary Figure 10 GPAT simulations: example of directional inconsistency model

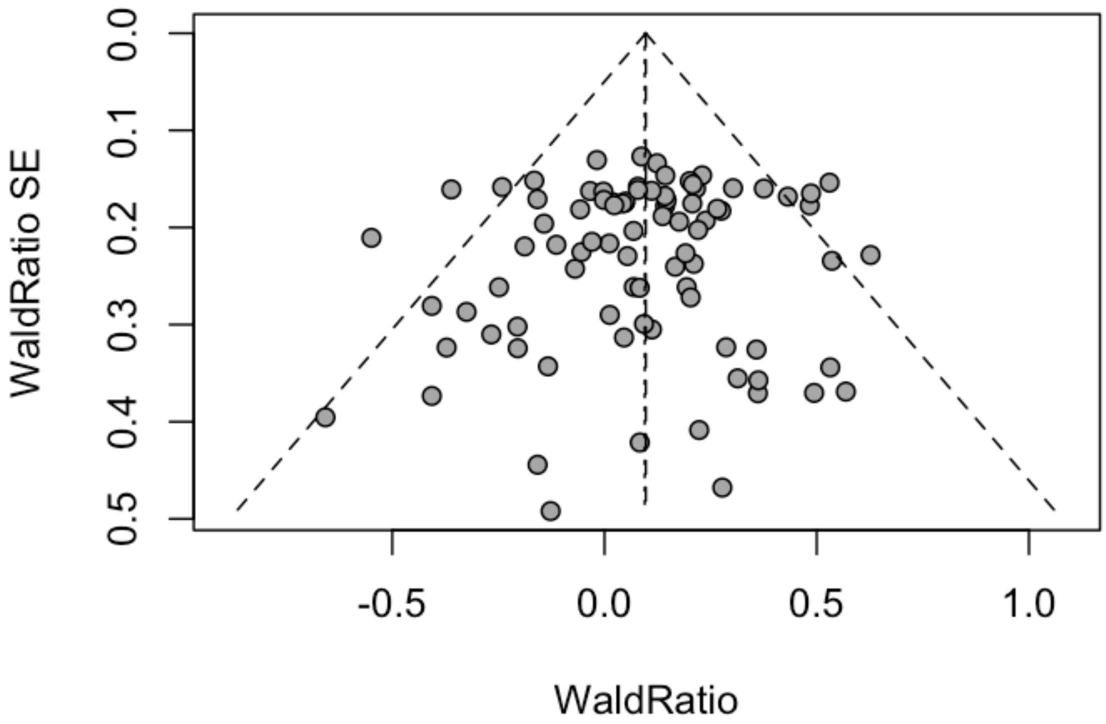

Each data point is the Wald ratio for a gene from simulation analyses under the directional inconsistency model. The dashed lines represent the fixed-effects (longer bars) and random-effects (shorter bars) meta-analysis estimates and the 95% confidence intervals. Estimates are presented on the linear scale.

Supplementary Tables

Supplementary Table 1 GPAT and MR for LDL-C and CHD

Estimator or parameter	GPAT		Mendelian randomization	
	OR (95% CI)	P-value	OR (95% CI)	P-value
Fixed-effects IVW	1.43 (1.31, 1.56)	2.1×10^{-16}	1.46 (1.43, 1.49)	3.4×10^{-244}
Heterogeneity	N/A	0.0003	N/A	3.8×10^{-138}
Random-effects IVW	1.52 (1.27, 1.81)	3.10×10^{-6}	1.46 (1.37, 1.56)	1.2×10^{-28}
Weighted median	1.31 (1.16, 1.47)	8.0×10^{-6}	1.35 (1.27, 1.44)	3.3×10^{-22}
Weighted mode	1.33 (1.19, 1.48)	1.8×10^{-6}	1.36 (1.29, 1.43)	1.4×10^{-28}
MR Egger (estimate)	1.37 (1.20, 1.56)	1.2×10^{-5}	1.45 (1.37, 1.53)	1.3×10^{-33}
MR Egger (intercept)	1.02 (0.99, 1.07)	0.19	1.00 (1.00, 1.00)	0.80

GPAT = Gene Perturbation Analysis for Transportability, MR = Mendelian randomization, LDL-C = low-density lipoprotein cholesterol, CHD = coronary heart disease, OR = Odds Ratio, CI = Confidence Interval, IVW = Inverse Variance Weighted.

GPAT and MR P-values are based on Z statistics from two-sided hypothesis tests with no multiple testing adjustment. Heterogeneity P-values are based on Cochran's Q statistic. MR Egger intercept P-values are based on t statistics from two-sided hypothesis tests with no multiple testing adjustment.

Supplementary Table 2 Gene-set enrichment estimates

Cellular phenotype	Number of genes	Human phenotype	% of screen hits with pLoF evidence	% of non-hits with pLoF evidence[1]	Enrichment ratio	Enrichment P-value
**Chondrocyte proliferation**	109	Adulthood height	16.50%	10.30%	1.6	0.049
**Insulin content**	522	Hba1c	4.60%	5.70%	0.8	0.31
		Type II diabetes (OR)	8.50%	7.40%	1.14	0.96
**Adipocyte differentiation**	27	Body mass index	18.50%	7.20%	2.57	0.06
		Waist circumference	14.80%	7.40%	1.99	0.27
		Body fat percentage	11.10%	7.20%	1.54	0.68
**Lysosomal cholesterol accumulation**	112	LDL-cholesterol	9.80%	6.50%	1.5	0.23

[1] all other protein-coding genes on autosomes plus chromosome X.

pLoF = predicted loss-of-function, OR = Odds Ratio.

P-values are based on Chi-squared statistics from two-sided hypothesis tests with no multiple testing adjustment.

Supplementary Table 3 GPAT estimates for lysosomal cholesterol accumulation and LDL-C

GPAT estimator or parameter	Estimate (95% CI)	P-value
Fixed-effects IVW	-0.006 (-0.010, -0.003)	0.0006
Heterogeneity	N/A	1.4×10^{-24}
Random-effects IVW	-0.010 (-0.020, 0.001)	0.06
Leave-one-out (*LDLR*) fixed-effects IVW	-0.004 (-0.008, -0.001)	0.02
Weighted median	-0.004 (-0.010, 0.002)	0.15
Weighted mode	-0.003 (-0.008, 0.002)	0.22
MR Egger (estimate)	-0.010 (-0.026, 0.006)	0.23
MR Egger (intercept)	0.010 (-0.031, 0.050)	0.64

GPAT = Gene Perturbation Analysis for Transportability, CI = Confidence Interval, IVW = Inverse Variance Weighted.

GPAT P-values are based on Z statistics from two-sided hypothesis tests with no multiple testing adjustment. Heterogeneity P-values are based on Cochran's Q statistic. MR Egger intercept P-values are based on t statistics from two-sided hypothesis tests with no multiple testing adjustment.

Supplementary Table 4 GPAT estimates for chondrocyte proliferation and standing height

GPAT estimator or parameter	Estimate (95% CI)	P-value
Fixed-effects IVW	-0.007 (-0.020, 0.006)	0.28
Heterogeneity	N/A	$4.9x10^{-15}$
Random-effects IVW	0.001 (-0.016, 0.039)	0.42
Weighted median	-0.020 (-0.042, 0.003)	0.09
Weighted mode	-0.017 (-0.038, 0.003)	0.1
MR Egger (estimate)	-0.014 (-0.086, 0.058)	0.71
MR Egger (intercept)	0.006 (-0.057, 0.070)	0.85

GPAT = Gene Perturbation Analysis for Transportability, CI = Confidence Interval, IVW = Inverse Variance Weighted.

GPAT P-values are based on Z statistics from two-sided hypothesis tests with no multiple testing adjustment. Heterogeneity P-values are based on Cochran's Q statistic. MR Egger intercept P-values are based on t statistics from two-sided hypothesis tests with no multiple testing adjustment.

Supplementary Table 5 GPAT estimates for adipocyte differentiation and adiposity-related phenotypes

GPAT estimator or parameter	Phenotype	Estimate (95% CI)	P-value
Fixed-effects IVW	Body mass index	0.007 (-0.006, 0.021)	0.3
	Waist circumference	0.013 (-0.001, 0.027)	0.07
	Body fat percentage	-0.007 (-0.020, 0.006)	0.28
Heterogeneity	Body mass index	N/A	0.03
	Waist circumference	N/A	0.01
	Body fat percentage	N/A	0.03
Random effects IVW	Body mass index	0.005 (-0.011, 0.021)	0.55
	Waist circumference	0.008 (-0.010, 0.026)	0.39
	Body fat percentage	0.011 (-0.016, 0.039)	0.42
Weighted Median	Body mass index	0.016 (-0.003, 0.034)	0.1
	Waist circumference	0.018 (-0.002, 0.038)	0.07
	Body fat percentage	-0.020 (-0.042, 0.003)	0.09
Weighted Mode	Body mass index	0.010 (-0.006, 0.026)	0.25
	Waist circumference	0.020 (0.003, 0.037)	0.03
	Body fat percentage	-0.017 (-0.038, 0.003)	0.1
MR Egger (estimate)	Body mass index	-0.036 (-0.100, 0.027)	0.27
	Waist circumference	-0.032 (-0.098, 0.035)	0.36
	Body fat percentage	-0.014 (-0.086, 0.058)	0.71
MR Egger (intercept)	Body mass index	0.072 (-0.028, 0.0172)	0.17
	Waist circumference	0.073 (-0.032, 0.178)	0.19
	Body fat percentage	0.006 (-0.057, 0.070)	0.85

GPAT = Gene Perturbation Analysis for Transportability, CI = Confidence Interval, IVW = Inverse Variance Weighted.

GPAT P-values are based on Z statistics from two-sided hypothesis tests with no multiple testing adjustment. Heterogeneity P-values are based on Cochran’s Q statistic. MR Egger intercept P-values are based on t statistics from two-sided hypothesis tests with no multiple testing adjustment.

Supplementary Table 6 GPAT estimates for insulin content and diabetes / Hba1c

GPAT estimator or parameter	Phenotype	Estimate (95% CI)	P-value
Fixed-effects IVW	Hba1c	-0.002 (-0.006, 0.003)	0.48
	T2D (OR)	1.00 (0.99, 1.02)	0.68
Heterogeneity	Hba1c	N/A	6.1×10^{-6}
	T2D	N/A	0.27
Random effects IVW	Hba1c	0.00 (-0.005, 0.005)	1
	T2D (OR)	1.01 (0.99, 1.02)	0.57
Weighted Median	Hba1c	-0.006 (-0.013, 0.002)	0.15
	T2D (OR)	0.99 (0.96, 1.02)	0.42
Weighted Mode	Hba1c	-0.006 (-0.014, 0.002)	0.13
	T2D (OR)	1.00 (0.96, 1.03)	0.76
MR Egger (estimate)	Hba1c	-0.009 (-0.021, 0.003)	0.15
	T2D (OR)	1.01 (0.97, 1.05)	0.56
MR Egger (intercept)	Hba1c	0.008 (-0.004, 0.021)	0.2
	T2D (OR)	0.99 (0.95, 1.03)	0.65

GPAT = Gene Perturbation Analysis for Transportability, CI = Confidence Interval, IVW = Inverse Variance Weighted, T2D = Type II Diabetes.

GPAT P-values are based on Z statistics from two-sided hypothesis tests with no multiple testing adjustment. Heterogeneity P-values are based on Cochran's Q statistic. MR Egger intercept P-values are based on t statistics from two-sided hypothesis tests with no multiple testing adjustment.

Supplementary Table 7 BioGRID ORCS data

ID	AUTHOR	CONDITION_NAME	*IN-VITRO* PHENOTYPE	CELL_TYPE	SCREEN_RATIONALE
1057	Patil A (2019)	CC-122 (Avadomide)	response to chemicals	Primary Effusion Lymphoma Cell Line	Increased drug resistance
1746	Thummuri D (2022)	Niraparib	response to chemicals	Pancreatic Adenocarcinom a Cell Line	Increased resistance to drug
2166	Koren I (2018)	-	protein/peptide accumulation	Embryonic Kidney Cell Line	Regulation of protein stability
2145	Koren I (2018)	-	protein/peptide accumulation	Embryonic Kidney Cell Line	Regulation of protein stability
2149	Koren I (2018)	-	protein/peptide accumulation	Embryonic Kidney Cell Line	Regulation of protein stability
2150	Koren I (2018)	-	protein/peptide accumulation	Embryonic Kidney Cell Line	Regulation of protein stability
2152	Koren I (2018)	-	protein/peptide accumulation	Embryonic Kidney Cell Line	Regulation of protein stability
2161	Koren I (2018)	-	protein/peptide accumulation	Embryonic Kidney Cell Line	Regulation of protein stability
2165	Koren I (2018)	-	protein/peptide accumulation	Embryonic Kidney Cell Line	Regulation of protein stability
2167	Koren I (2018)	-	protein/peptide accumulation	Embryonic Kidney Cell Line	Regulation of protein stability
1060	Patil A (2019)	Pomalidomide	response to chemicals	Primary Effusion Lymphoma Cell Line	Increased drug resistance
1061	Patil A (2019)	Pomalidomide	response to chemicals	Primary Effusion Lymphoma Cell Line	Increased drug resistance
2147	Koren I (2018)	-	protein/peptide accumulation	Embryonic Kidney Cell Line	Regulation of protein stability
1744	Thummuri D (2022)	Gemcitabine	response to chemicals	Pancreatic Adenocarcinom a Cell Line	Increased drug resistance
2146	Koren I (2018)	-	protein/peptide accumulation	Embryonic Kidney Cell Line	Regulation of protein stability
2159	Koren I (2018)	-	protein/peptide accumulation	Embryonic Kidney Cell Line	Regulation of protein stability
2160	Koren I (2018)	-	protein/peptide accumulation	Embryonic Kidney Cell Line	Regulation of protein stability
1056	Patil A (2019)	CC-122 (Avadomide)	response to chemicals	Primary Effusion Lymphoma Cell Line	Increased drug resistance
2153	Koren I (2018)	-	protein/peptide accumulation	Embryonic Kidney Cell Line	Regulation of protein stability
2156	Koren I (2018)	-	protein/peptide accumulation	Embryonic Kidney Cell Line	Regulation of protein stability
2157	Koren I (2018)	-	protein/peptide accumulation	Embryonic Kidney Cell Line	Regulation of protein stability
2164	Koren I (2018)	-	protein/peptide accumulation	Embryonic Kidney Cell Line	Regulation of protein stability
2020	Trimarco JD (2022)	Virus: Influenza B virus (B/Yamagata/16/1988)	response to virus	Lung Cancer Cell Line	Increased resistance to virus
2148	Koren I (2018)	-	protein/peptide accumulation	Embryonic Kidney Cell Line	Regulation of protein stability
2154	Koren I (2018)	-	protein/peptide accumulation	Embryonic Kidney Cell Line	Regulation of protein stability
2155	Koren I (2018)	-	protein/peptide accumulation	Embryonic Kidney Cell Line	Regulation of protein stability
2158	Koren I (2018)	-	protein/peptide accumulation	Embryonic Kidney Cell Line	Regulation of protein stability
2162	Koren I (2018)	-	protein/peptide accumulation	Embryonic Kidney Cell Line	Regulation of protein stability

1213	Ko T (2019)	Cisplatin	response to chemicals	Melanoma Cell Line	Increased drug resistance
1743	Thummuri D (2022)	Gemcitabine	response to chemicals	Pancreatic Adenocarcinoma Cell Line	Increased sensitivity to drug
2168	Koren I (2018)	-	protein/peptide accumulation	Embryonic Kidney Cell Line	Regulation of protein stability
2163	Koren I (2018)	-	protein/peptide accumulation	Embryonic Kidney Cell Line	Regulation of protein stability
1059	Patil A (2019)	Lenalidomide	response to chemicals	Primary Effusion Lymphoma Cell Line	Increased drug resistance
1574	Jia R (2019)	-	protein/peptide accumulation	Glioma Cell Line	Regulators of autophagy
1748	Thummuri D (2022)	5-fluorouracil	response to chemicals	Pancreatic Adenocarcinoma Cell Line	Increased drug resistance
1569	Barghout SH (2021)	TAK-243	response to chemicals	Acute Myeloid Leukemia Cell Line	Increased drug resistance
1745	Thummuri D (2022)	Niraparib	response to chemicals	Pancreatic Adenocarcinoma Cell Line	Increased sensitivity to drug
1566	Jia R (2021)	-	protein/peptide accumulation	Glioma Cell Line	Regulators of LC3B
1046	Kabir S (2019)	AZD5991 (MCL1i)	response to chemicals	Lung Squamous Cell Carcinoma Cell Line	Gene inhibition increases drug sensitivity
1058	Patil A (2019)	Lenalidomide	response to chemicals	Primary Effusion Lymphoma Cell Line	Increased drug resistance
1132	Labeau A (2020)	Virus: Dengue virus 2 Jamaica/1409/1983	response to virus	Chronic Myelogenous Leukemia Cell Line	Increased resistance to virus
1390	Park JS (2019)	Chlamydia trachomatis	response to bacteria	Colonic Adenocarcinoma Cell Line	Genes required for C. trachomatis invasion
1618	Morita K (2018)	-	protein/peptide accumulation	Embryonic Kidney Cell Line	Regulators of autophagy
1686	Mimura K (1970)	-	protein/peptide accumulation	Embryonic Kidney Cell Line	Negative regulators of autophagy
1747	Thummuri D (2022)	5-fluorouracil	response to chemicals	Pancreatic Adenocarcinoma Cell Line	Increased sensitivity to drug
2202	Heo JM (2019)	antimycin A/oligomycin A	protein transport	Cervical Adenocarcinoma Cell Line	Negative regulators of mitophagic flux
1018	Chang SJ (2019)	Typhoid Toxin	response to toxin	Embryonic Kidney Cell Line	Increased resistance to toxin
1575	Jia R (2019)	-	protein/peptide accumulation	Glioma Cell Line	Regulators of LC3B ubiquitination
1212	Ko T (2019)	Cisplatin	response to chemicals	Melanoma Cell Line	Increased sensitivity to drug
1897	Oreskovic E (2022)	-	protein/peptide accumulation	Mammary Epithelial Cell Line	Negative regulators of PD-L1 expression
1898	Oreskovic E (2022)	Interferon gamma	protein/peptide accumulation	Mammary Epithelial Cell Line	Negative regulators of PD-L1 expression in the presence of IFNgamma
1019	Chang SJ (2019)	Typhoid Toxin	response to toxin	Embryonic Kidney Cell Line	Increased resistance to toxin
1568	Barghout SH (2021)	TAK-243	response to chemicals	Acute Myeloid Leukemia Cell Line	Increased drug resistance
2204	Heo JM (2019)	antimycin A/oligomycin A	protein transport	Cervical Adenocarcinoma Cell Line	Positive regulators of mitophagic flux

1451	Thomsen EA (2020)	Rituximab	response to chemicals	Diffuse Large B-cell Lymphoma Cell	Increased resistance to chimeric monoclonal antibody
2206	Heo JM (2019)	antimycin A/oligomycin A	protein transport	Cervical Adenocarcinoma Cell Line	Positive regulators of mitophagic flux
1660	Gao S (2021)	-	cell proliferation	Colonic Cancer Cell Line	Cell-essential genes
2205	Heo JM (2019)	antimycin A/oligomycin A	protein transport	Cervical Adenocarcinoma Cell Line	Positive regulators of mitophagic flux
1426	van den Boomen DJH (2020)	-	protein/peptide accumulation	Cervical Adenocarcinoma Cell Line	Cellular cholesterol level maintenance genes
1045	Kabir S (2019)	AZD5576 (CDK9i)	response to chemicals	Lung Squamous Cell Carcinoma Cell Line	Gene inhibition increases drug sensitivity
2203	Heo JM (2019)	antimycin A/oligomycin A	protein transport	Cervical Adenocarcinoma Cell Line	Negative regulators of mitophagic flux
1425	van den Boomen DJH (2020)	-	protein/peptide accumulation	Cervical Adenocarcinoma Cell Line	Cellular cholesterol level maintenance genes
1150	Han J (2018)	Virus: Influenza A virus (A/Viet Nam/1203/2004(H5N1))	response to virus	Lung Cancer Cell Line	Increased resistance to virus
1661	Gao S (2021)	-	cell proliferation	Colonic Cancer Cell Line	Cell-essential genes
1524	Sharon DM (2020)	Virus: Influenza A (A/Puerto Rico/8/1934(H1N1)	response to virus	Embryonic Kidney Cell Line	Viral restriction factors
1355	Hinze L (2019)	Aspariginase	response to chemicals	Lymphoma or Leukaemia Cell Line	Increased sensitivity to drug
1429	Holmes DL (2020)	Virus: Human gammaherpesvirus 8 (Kaposi's sarcoma-associated herpesvirus KSHV)	response to virus	microvascular endothelial cell line	Decreased resistance to virus
1480	Zhang H (2020)	Salmonella enterica serovar Typhimurium	response to bacteria	Colonic Adenocarcinoma Cell Line	Increased resistance to bacteria
1801	Khan DH (2020)	-	cell proliferation	Acute Myeloid Leukemia Cell Line	Cell-essential genes
1479	Zhang H (2020)	Salmonella enterica serovar Typhimurium	response to bacteria	Colonic Adenocarcinoma Cell Line	Increased resistance to bacteria
1725	Condon KJ (2021)	-	protein/peptide accumulation	Embryonic Kidney Cell Line	mTORC1 activation
1713	Lee DH (2021)	Cultured in the absence of GM-CSF	cell proliferation	Erythroleukemia Cell Line	Growth restrictive genes
1561	Kulsuptrakul J (2021)	Virus: Human hepatitis A virus HM175	response to virus	Huh-7 Cell	Increased resistance to virus
1115	Xu S (2019)	Gemcitabine	response to chemicals	Gall Bladder Cancer Cell Line	Increased drug resistance
1214	Ko T (2019)	-	cell proliferation	Melanoma Cell Line	Cell-essential genes
1193	Fei T (2019)	-	cell proliferation	Prostate Cancer Cell Line	Cell-essential genes
1220	Wang R (2020)	Virus: SARS-CoV-2 (Severe acute respiratory syndrome coronavirus 2)	response to virus	Huh-7 Cell	Increased resistance to virus
371	Cheng J (2017)	-	cell proliferation	Cancer Cell Line	Cell-essential genes
1149	Han J (2018)	Virus: Influenza A virus (A/Viet Nam/1203/2004(H5N1))	response to virus	Lung Cancer Cell Line	Increased resistance to virus

1298	Chai AWY (2020)	-	cell proliferation	Oral Squamous Cell Carcinoma Cell Line	Cell-essential genes
1192	Fei T (2019)	-	cell proliferation	Breast Cancer Cell Line	Cell-essential genes
1017	Flint M (2019)	Virus: Ebola virus (Mayinga strain)	response to virus	Huh-7 Cell	Increased resistance to virus
1297	Chai AWY (2020)	-	cell proliferation	Oral Squamous Cell Carcinoma Cell Line	Cell-essential genes
1109	Shifrut E (2018)	-	protein/peptide accumulation	Regulatory T cell	T cell proliferation inhibition genes
1221	Wang R (2020)	Virus: HCoV-OC43 (Human coronavirus OC43)	response to virus	Huh-7 Cell	Increased resistance to virus
1222	Wang R (2020)	Virus: HCoV-229E (Human coronavirus 229E)	response to virus	Huh-7 Cell	Increased resistance to virus
1110	Shifrut E (2018)	CGS-21680	protein/peptide accumulation	Regulatory T cell	Increased T cell resistance to immunosuppressive factor
1302	Chai AWY (2020)	-	cell proliferation	Oral Squamous Cell Carcinoma Cell Line	Cell-essential genes
1148	Han J (2018)	Virus: Influenza A virus (A/Viet Nam/1203/2004(H5N1))	response to virus	Lung Cancer Cell Line	Increased resistance to virus
1361	Nechiporuk T (2019)	Venetoclax	response to chemicals	Acute Myeloid Leukemia Cell Line	Increased drug resistance
1362	Nechiporuk T (2019)	Venetoclax	response to chemicals	Acute Myeloid Leukemia Cell Line	Increased drug resistance
1573	Jia R (2019)	-	protein/peptide accumulation	Glioma Cell Line	Regulators of autophagy
1209	Dukhovny A (2019)	Virus: Zika virus	response to virus	Hepatoma Cell Line	Activation increases resistance to the virus
1434	Trinh MN (2020)	-	protein/peptide accumulation	Immortal Cell Line	LDL-derived cholesterol transport to the ER genes
1363	Nechiporuk T (2019)	Venetoclax	response to chemicals	Acute Myeloid Leukemia Cell Line	Increased drug resistance
1210	Dukhovny A (2019)	Virus: Zika virus	response to virus	Hepatoma Cell Line	Activation increases resistance to the virus
1308	Chai AWY (2020)	-	cell proliferation	Cancer Cell Line	Cell-essential genes
1293	Chai AWY (2020)	-	cell proliferation	Oral Squamous Cell Carcinoma Cell Line	Cell-essential genes
1310	Chai AWY (2020)	-	cell proliferation	Oral Squamous Cell Carcinoma Cell Line	Cell-essential genes
1301	Chai AWY (2020)	-	cell proliferation	Oral Squamous Cell Carcinoma Cell Line	Cell-essential genes
1669	Giovannucci TA (2021)	CBK77	response to chemicals	Melanoma Cell Line	Increased drug resistance
1296	Chai AWY (2020)	-	cell proliferation	Oral Squamous Cell Carcinoma Cell Line	Cell-essential genes
1294	Chai AWY (2020)	-	cell proliferation	Oral Squamous Cell Carcinoma Cell Line	Cell-essential genes
1304	Chai AWY (2020)	-	cell proliferation	Oral Squamous Cell Carcinoma Cell Line	Cell-essential genes
1300	Chai AWY (2020)	-	cell proliferation	Oral Squamous Cell Carcinoma Cell Line	Cell-essential genes

1292	Chai AWY (2020)	-	cell proliferation	Oral Squamous Cell Carcinoma Cell Line	Cell-essential genes
1311	Chai AWY (2020)	-	cell proliferation	Oral Squamous Cell Carcinoma Cell Line	Cell-essential genes
1295	Chai AWY (2020)	-	cell proliferation	Oral Squamous Cell Carcinoma Cell Line	Cell-essential genes
1306	Chai AWY (2020)	-	cell proliferation	Tongue Cancer Cell Line	Cell-essential genes
1299	Chai AWY (2020)	-	cell proliferation	Oral Squamous Cell Carcinoma Cell Line	Cell-essential genes
1303	Chai AWY (2020)	-	cell proliferation	Oral Squamous Cell Carcinoma Cell Line	Cell-essential genes
1307	Chai AWY (2020)	-	cell proliferation	Tongue Cancer Cell Line	Cell-essential genes
1312	Chai AWY (2020)	-	cell proliferation	Oral Squamous Cell Carcinoma Cell Line	Cell-essential genes
1309	Chai AWY (2020)	-	cell proliferation	Oral Squamous Cell Carcinoma Cell Line	Cell-essential genes
1305	Chai AWY (2020)	-	cell proliferation	Oral Squamous Cell Carcinoma Cell Line	Cell-essential genes
1430	Holmes DL (2020)	Virus: Human gammaherpesvirus 8 (Kaposi's sarcoma-associated herpesvirus KSHV)	response to virus	microvascular endothelial cell line	Decreased resistance to virus

Supplementary Table 8 Hypothesis-free GPAT estimates

ID	human *in-vivo* phenotype (UK Biobank)	GPAT_beta_fe	GPAT_se_fe	GPAT_pval_fe	GPAT_n_genes	AUTHOR	*IN-VITRO* PHENOTYPE	CELL_TYPE
1713	Lymphocyte percentage	-0.033183794	0.004327779	1.75E-14	101	Lee DH (2021)	cell proliferation	Erythroleukemia Cell Line
1115	Triglycerides	-0.020062749	0.002663716	5.00E-14	189	Xu S (2019)	response to chemicals	Gall Bladder Cancer Cell Line
1193	Erythrocyte distribution width	-0.012502658	0.001812529	5.28E-12	236	Fei T (2019)	cell proliferation	Prostate Cancer Cell Line
1295	Erythrocyte distribution width	-0.004769437	0.000725751	4.97E-11	1069	Chai AWY (2020)	cell proliferation	Oral Squamous Cell Carcinoma Cell Line
1309	Erythrocyte distribution width	-0.003702691	0.000569885	8.18E-11	1256	Chai AWY (2020)	cell proliferation	Oral Squamous Cell Carcinoma Cell Line
1312	Erythrocyte distribution width	-0.003837119	0.000595239	1.15E-10	1226	Chai AWY (2020)	cell proliferation	Oral Squamous Cell Carcinoma Cell Line
1299	Erythrocyte distribution width	-0.006067813	0.000948781	1.60E-10	1112	Chai AWY (2020)	cell proliferation	Oral Squamous Cell Carcinoma Cell Line
1292	Erythrocyte distribution width	-0.003467797	0.000544222	1.87E-10	1030	Chai AWY (2020)	cell proliferation	Oral Squamous Cell Carcinoma Cell Line
1311	Erythrocyte distribution width	-0.003098554	0.000486882	1.96E-10	1077	Chai AWY (2020)	cell proliferation	Oral Squamous Cell Carcinoma Cell Line
1304	Erythrocyte distribution width	-0.003332849	0.000530483	3.33E-10	1014	Chai AWY (2020)	cell proliferation	Oral Squamous Cell Carcinoma Cell Line
1294	Erythrocyte distribution width	-0.004668849	0.000753768	5.86E-10	998	Chai AWY (2020)	cell proliferation	Oral Squamous Cell Carcinoma Cell Line
1306	Erythrocyte distribution width	-0.004564413	0.000740729	7.18E-10	1106	Chai AWY (2020)	cell proliferation	Tongue Cancer Cell Line
1296	Erythrocyte distribution width	-0.007376092	0.00122817	1.90E-09	967	Chai AWY (2020)	cell proliferation	Oral Squamous Cell Carcinoma Cell Line
1713	Neutrophil percentage	0.025322185	0.004337337	5.28E-09	101	Lee DH (2021)	cell proliferation	Erythroleukemia Cell Line
1300	Erythrocyte distribution width	-0.006313163	0.001088772	6.69E-09	1017	Chai AWY (2020)	cell proliferation	Oral Squamous Cell Carcinoma Cell Line
1308	Erythrocyte distribution width	-0.00621951	0.001075424	7.32E-09	868	Chai AWY (2020)	cell proliferation	Cancer Cell Line
1303	Erythrocyte distribution width	-0.004239245	0.000736121	8.47E-09	1139	Chai AWY (2020)	cell proliferation	Oral Squamous Cell Carcinoma Cell Line
2167	Standing height	-0.100661503	0.01754892	9.69E-09	2	Koren I (2018)	protein/peptide accumulation	Embryonic Kidney Cell Line
2165	Standing height	-0.110539951	0.01927406	9.74E-09	2	Koren I (2018)	protein/peptide accumulation	Embryonic Kidney Cell Line
1305	Erythrocyte distribution width	-0.003279897	0.000583607	1.91E-08	1270	Chai AWY (2020)	cell proliferation	Oral Squamous Cell Carcinoma Cell Line
2166	Standing height	-0.097239051	0.017326551	2.00E-08	1	Koren I (2018)	protein/peptide accumulation	Embryonic Kidney Cell Line
1192	Erythrocyte distribution width	-0.010345389	0.00185555	2.47E-08	446	Fei T (2019)	cell proliferation	Breast Cancer Cell Line
1310	Erythrocyte distribution width	-0.005263681	0.000945386	2.58E-08	927	Chai AWY (2020)	cell proliferation	Oral Squamous Cell Carcinoma Cell Line

1301	Erythrocyte distribution width	-0.004461027	0.000801823	2.64E-08	924	Chai AWY (2020)	cell proliferation	Oral Squamous Cell Carcinoma Cell Line
1307	Erythrocyte distribution width	-0.004246981	0.000779377	5.06E-08	1183	Chai AWY (2020)	cell proliferation	Tongue Cancer Cell Line
1293	Erythrocyte distribution width	-0.006334204	0.001202816	1.39E-07	890	Chai AWY (2020)	cell proliferation	Oral Squamous Cell Carcinoma Cell Line
1297	Erythrocyte distribution width	-0.013953633	0.002656709	1.50E-07	473	Chai AWY (2020)	cell proliferation	Oral Squamous Cell Carcinoma Cell Line
1713	Neutrophil percentage	0.020944527	0.004318194	1.23E-06	101	Lee DH (2021)	cell proliferation	Erythroleukemia Cell Line
1292	Cystatin	-0.002576637	0.00054201	2.00E-06	1028	Chai AWY (2020)	cell proliferation	Oral Squamous Cell Carcinoma Cell Line
1661	Erythrocyte distribution width	-0.014491127	0.003080597	2.55E-06	98	Gao S (2021)	cell proliferation	Colonic Cancer Cell Line
1573	Platelet count	-0.006532259	0.001430047	4.93E-06	79	Jia R (2019)	protein/peptide accumulation	Glioma Cell Line

Supplementary Table 9 GPAT simulation results (N = 100)

**Model**	*GPAT IVW*<br><br>Ratio of estimate scaled against simulated effect (95% CI)	*MR Egger*<br><br>Ratio of estimate scaled against simulated effect (95% CI)	*MR Egger Intercept*<br><br>Median P-value
True causal estimate[1]	1	1	N/A
Baseline	0.56 (0.54, 0.58)	0.44 (0.38, 0.49)	0.33
Balanced pleiotropy	0.59 (0.54, 0.64)	0.54 (0.37, 0.70)	0.45
Unbalanced pleiotropy	2.65 (2.59, 2.71)	0.51 (0.33, 0.70)	0.007
Phenotypic pleiotropy	1.73 (1.70, 1.76)	1.39 (1.33, 1.45)	0.16
Directional inconsistency	0.36 (0.34, 0.38)	0.27 (0.22, 0.33)	0.30

[1] all estimates are expected to be biased downwards by ~40% due to not controlling for differences in magnitude of effect between experimental and LoF perturbations.

GPAT = Gene Perturbation Analysis for Transportability, CI = Confidence Interval, IVW = Inverse Variance Weighted.

MR Egger intercept P-values are based on t statistics from two-sided hypothesis tests with no multiple testing adjustment.

Supplementary Table 10 UK Biobank phenotypes

**Human *in-vivo* phenotype (UKB)**	**UKB Data Showcase ID**
Standing height	50
BMI	21001
Waist circumference	48
Whole body fat mass	23100
FEV1/FVC ratio	20258
Leukocyte count	30000
Erythrocyte count	30010
Platelet count	30080
Lymphocyte count	30120
Monocyte count	30130
Neutrophil count	30140
Eosinophil count	30150
Basophil count	30160
Reticulocyte count	30240
Lymphocyte percentage	30180
Monocyte percentage	30190
Neutrophil percentage	30200
Eosinophil percentage	30210
Basophil percentage	30220
Reticulocyte percentage	30240
Haemoglobin concentration	30020
Haemoglobin percentage	30030
Mean corpuscular volume	30040
Mean corpuscular haemoglobin	30050
Mean corpuscular haemoglobin concentration	30060
Erthrocyte distribution width	20070
Platelet crit	30090
Mean platelet thrombocyte volume	30100
Platelet distribution width	30110
Mean reticulocyte volume	30260
Mean sphered cell volume	30270
Immature reticulocyte fraction	30280
High light scatter reticulocyte percentage	30290
High light scatter reticulocyte count	30300
Albumin	30600
Alanine aminotransferase	30620
Alkaline phosphatase	30610
Apolipoprotein A	30630
Apolipoprotein B	30640

Aspartate aminotransferase	30650
Bilirubin (direct)	30660
Calcium	30680
Cholesterol	30690
Creatinine	30700
CRP	30710
Cystatin	30720
Gamma glutamyltransferase	30730
Glucose	30740
Hba1c	30750
HDL-cholesterol	30760
IGF-1	30770
LDL-cholesterol (direct)	30780
Lipoprotein A	30790
Oestradiol	30800
Phosphate	30810
Rheumatoid factor	30820
SHBG	30830
Testosterone	30850
Total bilirubin	30840
Total protein	30860
Triglycerides	30870
Urate	30880
Urea	30670
Vitamin D	30890
Creatinine in Urine	30510
Non-albumin protein	30860, 30600
Potassium in urine	30520
Microalbumin in urine	30500
Sodium in urine	30530

UKB = UK Biobank.
