# Supplementary material for: Evaluating transportability of in vitro cellular models to in vivo human phenotypes using gene perturbation data
Source: Nat Commun. 2025 Dec 13;17:513. doi: 10.1038/s41467-025-67199-1 (PMC12804867; doi:10.1038/s41467-025-67199-1)
Supplement: Supplementary file 2 — Reporting Summary [file 41467_2025_67199_MOESM2_ESM.pdf]

nature portfolio

Corresponding author(s): LAURENCE HOWE

Last updated by author(s): Nov 5, 2025

Reporting Summary

Nature Portfolio wishes to improve the reproducibility of the work that we publish. This form provides structure for consistency and transparency in reporting. For further information on Nature Portfolio policies, see our Editorial Policies and the Editorial Policy Checklist.

Statistics

For all statistical analyses, confirm that the following items are present in the figure legend, table legend, main text, or Methods section.

n/a	Confirmed	
☐	☒	The exact sample size (*n*) for each experimental group/condition, given as a discrete number and unit of measurement
☐	☒	A statement on whether measurements were taken from distinct samples or whether the same sample was measured repeatedly
☐	☒	The statistical test(s) used AND whether they are one- or two-sided<br>*Only common tests should be described solely by name; describe more complex techniques in the Methods section.*
☐	☒	A description of all covariates tested
☐	☒	A description of any assumptions or corrections, such as tests of normality and adjustment for multiple comparisons
☐	☒	A full description of the statistical parameters including central tendency (e.g. means) or other basic estimates (e.g. regression coefficient) AND variation (e.g. standard deviation) or associated estimates of uncertainty (e.g. confidence intervals)
☐	☒	For null hypothesis testing, the test statistic (e.g. *F*, *t*, *r*) with confidence intervals, effect sizes, degrees of freedom and *P* value noted<br>*Give P values as exact values whenever suitable.*
☒	☐	For Bayesian analysis, information on the choice of priors and Markov chain Monte Carlo settings
☒	☐	For hierarchical and complex designs, identification of the appropriate level for tests and full reporting of outcomes
☐	☒	Estimates of effect sizes (e.g. Cohen's *d*, Pearson's *r*), indicating how they were calculated

Our web collection on statistics for biologists contains articles on many of the points above.

Software and code

Policy information about availability of computer code

Data collection	No software was used for data collection.
Data analysis	Standard functions from the TwoSampleMR (0.6.20) and metagen (8.1-0) R packages were used for all empirical GPAT analyses and visualizations as described in the manuscript. Custm R code used for GPAT simulations has been deposited in a public repository (https://zenodo.org/records/17534798).

For manuscripts utilizing custom algorithms or software that are central to the research but not yet described in published literature, software must be made available to editors and reviewers. We strongly encourage code deposition in a community repository (e.g. GitHub). See the Nature Portfolio guidelines for submitting code & software for further information.

Data

Policy information about availability of data

All manuscripts must include a data availability statement. This statement should provide the following information, where applicable:

- Accession codes, unique identifiers, or web links for publicly available datasets
- A description of any restrictions on data availability
- For clinical datasets or third party data, please ensure that the statement adheres to our policy

The experimental data used in this study are publicly available. In-vitro perturbation data were downloaded from the supplementary material of publications (as described and referenced in the manuscript) and from the BioGRID Open Repository of CRISPR Screens (ORCS) (version 1.1.16, May 2024) website https://

orcs.thebiogrid.org/.
The human pLoF burden association data used in this study were derived from individual participant data from the UK Biobank. Phenotypic and genotypic data from UK Biobank are available to researchers via an application https://www.ukbiobank.ac.uk/enable-your-research/apply-for-access. Phenome-wide pLoF burden data (same dataset, similar variant masks) from the UK Biobank WGS dataset are in the AstraZeneca PheWAS browser (https://azphewas.com/).

Research involving human participants, their data, or biological material

Policy information about studies with human participants or human data. See also policy information about sex, gender (identity/presentation), and sexual orientation and race, ethnicity and racism.

Reporting on sex and gender	N/A
Reporting on race, ethnicity, or other socially relevant groupings	Ancestry (used to restrict burden analyses to non-finnish Europeans) was defined based on genomic principal components.
Population characteristics	UK Biobank is a general population cohort of individuals recruited between the ages of 40 and 60.
Recruitment	UK Biobank recruited 500,000 people across the country aged between 40 and 69 years from 2006 to 2010. These half a million participants agreed to have their health followed so scientists across the globe can help improve the prevention, diagnosis and treatment of a wide range of diseases.
Ethics oversight	UK Biobank has approval from the North West Multi-centre Research Ethics Committee (MREC) as a Research Tissue Bank (RTB) approval.

Note that full information on the approval of the study protocol must also be provided in the manuscript.

Field-specific reporting

Please select the one below that is the best fit for your research. If you are not sure, read the appropriate sections before making your selection.

☒ Life sciences ☐ Behavioural & social sciences ☐ Ecological, evolutionary & environmental sciences

For a reference copy of the document with all sections, see nature.com/documents/nr-reporting-summary-flat.pdf

Life sciences study design

All studies must disclose on these points even when the disclosure is negative.

Sample size	For burden tests we used the maximum sample-size of non-Finnish Europeans in UK Biobank after quality control exclusions on the WGS data. This dataset includes hundreds of thousands of individuals and so is currently the largest available dataset for GPAT analyses.
Data exclusions	We analysed only non-Finnish Europeans to reduce bias from population stratification which may inflate test statistic estimates. We also excluded samples failing QC.
Replication	Replication is not applicable at present as we used the largest available dataset of whole genome sequence data.
Randomization	Not relevant to this study design based on population data.
Blinding	Not relevant as no randomization.

Reporting for specific materials, systems and methods

We require information from authors about some types of materials, experimental systems and methods used in many studies. Here, indicate whether each material, system or method listed is relevant to your study. If you are not sure if a list item applies to your research, read the appropriate section before selecting a response.

Materials & experimental systems

n/a	Involved in the study
☒	☐ Antibodies
☒	☐ Eukaryotic cell lines
☒	☐ Palaeontology and archaeology
☒	☐ Animals and other organisms
☒	☐ Clinical data
☒	☐ Dual use research of concern
☒	☐ Plants

Methods

n/a	Involved in the study
☒	☐ ChIP-seq
☒	☐ Flow cytometry
☒	☐ MRI-based neuroimaging

Plants

Seed stocks

Report on the source of all seed stocks or other plant material used. If applicable, state the seed stock centre and catalogue number. If plant specimens were collected from the field, describe the collection location, date and sampling procedures.

Novel plant genotypes

Describe the methods by which all novel plant genotypes were produced. This includes those generated by transgenic approaches, gene editing, chemical/radiation-based mutagenesis and hybridization. For transgenic lines, describe the transformation method, the number of independent lines analyzed and the generation upon which experiments were performed. For gene-edited lines, describe the editor used, the endogenous sequence targeted for editing, the targeting guide RNA sequence (if applicable) and how the editor was applied.

Authentication

Describe any authentication procedures for each seed stock used or novel genotype generated. Describe any experiments used to assess the effect of a mutation and, where applicable, how potential secondary effects (e.g. second site T-DNA insertions, mosiacism, off-target gene editing) were examined.
